# The European Medicines Agency’s scientific opinion on oral fexinidazole for human African trypanosomiasis

**DOI:** 10.1371/journal.pntd.0007381

**Published:** 2019-06-27

**Authors:** Eric Pelfrene, Martin Harvey Allchurch, Nsengi Ntamabyaliro, Victoria Nambasa, Fátima V. Ventura, Nithyanandan Nagercoil, Marco Cavaleri

**Affiliations:** 1 Office of Anti-infectives and Vaccines, Human Medicines Evaluation Division, European Medicines Agency (EMA), Amsterdam, The Netherlands; 2 International Affairs Division, European Medicines Agency (EMA), Amsterdam, The Netherlands; 3 Clinical Pharmacology Unit, University of Kinshasa, Democratic Republic of the Congo; 4 Uganda National Drug Authority, Kampala, Uganda; 5 Medicines Evaluation Department, National Authority of Medicines and Health Products (INFARMED), Lisbon, Portugal; 6 Licensing Division, Medicines and Healthcare products Regulatory Agency (MHRA), London, United Kingdom; Swiss Tropical and Public Health Institute, SWITZERLAND

## Introduction

The European Medicines Agency‘s scientific Committee for Medicinal Products for Human Use (CHMP) issued a positive opinion on 15 November 2018 for fexinidazole tablets for the treatment of human African trypanosomiasis (HAT; sleeping sickness) caused by the protozoon *Trypanosoma brucei gambiense* (g-HAT) subspecies. This is a life-threatening neglected tropical disease transmitted by tsetse flies in western and central Africa. *T*. *b*. *gambiense* causes 97% of HAT cases and is responsible for the chronic form of the disease, which evolves toward a fatal outcome in 2 to 3 years following infection if left untreated [1]. The CHMP opinion on fexinidazole was adopted under a unique evaluation process specifically designed to provide a benefit–risk evaluation for medicines relevant to populations living in regions outside the European Union (EU) [2]. The scientific assessment, under Article 58 of Regulation (EC) No 726/2004 was conducted in cooperation with the World Health Organization (WHO), in particular with the WHO Department of Control of Neglected Tropical Diseases and involved national competent authorities from the Democratic Republic of the Congo (DRC) and Uganda, thereby facilitating registration in target countries.

## Benefit–risk evaluation (salient aspects)

Fexinidazole, a nitroimidazole derivative, represents the first exclusively oral regimen for the treatment of both haemolymphatic [stage 1] and meningoencephalitic [stage 2] stages of g-HAT in adults and adolescents as well as in children aged 6 years and above and weighing at least 20 kg. The opinion was mainly based on the favorable results obtained in the pivotal trial (DNDiFEX 004) and supporting evidence from 2 additional noncomparative open-label trials: one conducted in adult patients (>15 years of age, *N* = 230), with less advanced g-HAT [stage 1 or early stage 2] (DNDiFEX 005), and the second, in children aged 6 to 15 years (*N* = 125), enrolled regardless of the infection stage (DNDiFEX 006). As previously reported, DNDiFEX 004 was a randomized, open-label, noninferiority study comparing fexinidazole against nifurtimox-eflornithine (NECT) reference therapy in 394 patients, aged 15 years and above, suffering from meningoencephalitic g-HAT [3, 4]. Although concluding on a lower efficacy estimate for fexinidazole as compared with NECT (91.2% versus 97.6%; −6.4%, 97.06% CI [−11.2 to −1.6]; *p* = 0·0029), the difference observed was within the prespecified noninferiority margin of −13%. A relatively lower efficacy of this magnitude was considered acceptable by the applicant on the grounds that it would be outweighed by an easier distribution and administration of the medicinal product in comparison with NECT, potentially allowing g-HAT patients quicker and wider access to treatment. The results gathered were largely driven by the subgroup of patients with a severe meningoencephalitic compromise, defined by >100 white blood cells (WBC) per μL of cerebrospinal fluid (CSF) for which fexinidazole showed 11.8% less favorable outcome compared with NECT (86.9% versus 98.7%); in the complementary subgroup of patients with ≤100 WBC per μL of CSF, the treatment success of fexinidazole was 98%, which was similar to the 95.9% success rate of NECT.

The CHMP convened a panel of independent experts from WHO and the affected region in September 2018 to advise on the potential role of fexinidazole in the treatment of both stages of g-HAT, taking into account the local conditions and the current changing disease epidemiology. The most contentious issue related to the feasibility at peripheral health facilities of reliably differentiating patients at a higher risk of failure with fexinidazole compared with NECT, from those who are expected to have similar benefit with either treatment. Despite attempts by the applicant (Sanofi-Aventis Groupe, France) to establish a correlation between signs and symptoms and the stage of HAT to avoid lumbar puncture in the identification of patients with advanced HAT, reliable clinical score-based predictive criteria could not be validated. Identification of clinical features potentially related with stage 2 g-HAT is subjective and affected by the sensitivity and skills of the clinicians and the nature of the patients. This is further corroborated by published reports in which inconsistency in the association of clinical features with high CSF-WBC counts is described [5, 6]. Further work is needed to find a reliable method or marker to replace lumbar puncture in the diagnosis of these patients. As such, enumeration of WBCs in the CSF is currently the best marker to predict the success or failure of the fexinidazole regimen. It was recommended that stage 2 patients with >100 WBC per μL of CSF should only be treated with fexinidazole if no other adequate treatment (e.g., NECT) is available or tolerated. This is a notable limitation of this treatment in the current context of targeted elimination of HAT as a public health problem by 2020 [7].

Another important aspect pertained to the best method of ensuring compliance given the prerequisite of a concurrent meal to ensure sufficient drug exposure and the propensity of vomiting following fexinidazole. It was considered that tablet administration under direct supervision of trained health staff was warranted to ensure compliance and administration of a full effective treatment course. In patients with risk of poor compliance to treatment, hospitalized administration of treatment is required. Given the risk of late relapse following fexinidazole therapy, it was also considered necessary that this risk should be communicated to patients and carers, and appropriate local arrangements should be made to allow an optimum 24-months follow-up after a treatment course. The main advantages and limitations of the therapeutic innovation are depicted in Box 1. Detailed considerations regarding the data evaluation are available on the Agency’s website [8].

**Box 1. Advantages and limitations of fexinidazole in treatment of gambiense HAT**

**Advantages**

- First exclusively oral regimen for the treatment of g-HAT
- Treatment effective in both haemolymphatic and meningoencephalitic disease stages
- Potential for increased access to treatment (easier and wider distribution in respect to the current reference therapies; no need for sophisticated healthcare infrastructure; lower implementation costs assumed)

**Limitations**

- Staging: Lumbar puncture cannot be systematically avoided; severe patients (with CSF-WBC > 100 /μL) should only be treated with fexinidazole if no other adequate treatment is available or tolerated.
- Compliance: Nausea and vomiting have been noted with fexinidazole administration. Drug absorption is dependent on ingestion of food; as such, trained health staff need to confirm that the patient is in fed condition as well as directly observe each drug intake.
  In patients with risk of poor compliance, as well as in children with a body weight lower than 35 kg, in patients with psychiatric disorders, and in the exceptional cases of severe patients treated with fexinidazole, hospitalized administration is warranted
- Relapses: Late relapse has been noted in the pivotal and supportive studies conducted with fexinidazole; all patients should have follow-up monitoring at recurrence of symptoms suggestive of HAT, at 12 months and up to 24 months after treatment completion with fexinidazole. Patients should be made aware of the risk of relapse after therapy and instructed to contact the healthcare staff in case of signs of relapse.

The Article 58 evaluation pathway (also known as EU-Medicines4all or EU-M4all) is gaining more acceptance from regulators in non-EU countries, pharmaceutical companies, and nongovernmental organizations active in global health. Despite slow initial uptake [9, 10], the number of applications and opinions has increased recently, as well as the number of national registrations in non-EU countries based on the CHMP opinions. We are convinced that it constitutes an important regulatory tool to facilitate regulators in low- and middle-income countries in providing access to safe, efficacious, and quality-assured medicines, including vaccines [2, 11]. It also provides a unique pathway to support both commercial and not-for-profit development of medicines to prevent or treat priority diseases together with advice from EMA, WHO, local regulators, and experts. This is most effectively demonstrated by the rapid national authorization of fexinidazole by the DRC authorities in December 2018 on the basis of the CHMP scientific assessment. As such, we welcome CHMP’s evaluation and positive scientific opinion on fexinidazole, providing an oral alternative to cure those affected by g-HAT and lending further support to the ultimate goal of eliminating this devastating vector-borne infection.
